# Systematical Screening of Intracellular Protein Targets of Polyphemusin-I Using *Escherichia coli* Proteome Microarrays

**DOI:** 10.3390/ijms22179158

**Published:** 2021-08-25

**Authors:** Pramod Shah, Chien-Sheng Chen

**Affiliations:** 1 Institute of Systems Biology and Bioinformatics, Department of Biomedical Sciences and Engineering, College of Health Sciences and Technology, National Central University, Jhongli 300, Taiwan; prokonp@gmail.com; 2 Department of Nutritional Science, Fu Jen Catholic University, New Taipei City 242, Taiwan; 3 Department of Food Safety/Hygiene and Risk Management, College of Medicine, National Cheng Kung University, Tainan 701, Taiwan

**Keywords:** polyphemusin-I, protein target, target identification, antimicrobial peptides (AMPs), protein microarray

## Abstract

With their wide repertoire of mechanisms, antimicrobial peptides (AMPs) are promising alternatives to fight against varied pathogenic microorganisms (bacteria, fungi, viruses, parasites, etc.). AMPs, novel components of the innate immune defense system, are secreted by all organisms. The aquatic environment represents a huge population and an enormous source of varied AMPs. Polyphemusin-I, a marine AMP isolated from hemocytes of an American horseshoe crab, possesses high antimicrobial activities. Studies on polyphemusin-I have verified the intracellular mechanisms of action, however, its intracellular targets are not yet explored. In this study, we employed *Escherichia coli* proteome microarrays to systematically screen the entire intracellular protein targets of polyphemusin-I. A total of 97 protein targets of polyphemusin-I were statistically analyzed from the quadruplicate *Escherichia coli* proteome microarrays assays. Among these identified protein targets, 56 proteins had cellular location inside the cell (i.e., cytoplasm), one in the plasma membrane, one in the periplasm and the rest 39 proteins had no specified cellular location. The bioinformatics analysis of these identified protein targets of polyphemusin-I in gene ontology (GO) enrichment category of molecular function revealed significant enrichment in nucleic acid related GO terms i.e., “RNA binding”, “nucleotide binding”, “nuclease activities”, “uracil DNA N-glycosylase activities” and others. Moreover, enrichment in GO category of biological process also depicted enrichment in nucleic acid related GO terms, such as “nucleic acid phosphodiester bond hydrolysis”, “deoxyribonucleotide metabolism”, and others. In accordance to GO enrichment analysis, protein families (PFAM) and Kyoto Encyclopedia of Genes and Genomes (KEGG) pathways enrichment analysis also showed significant enrichment in nucleic acid terms. These enrichment results suggest that polyphemusin-I targets nucleic acid-associated proteins. Furthermore, to provide a comprehensive study, we compared the identified protein targets of polyphemusin-I with previously identified protein targets of four AMPs (P-Der, Lfcin B, PR-39, and Bac 7) using *Escherichia coli* proteome microarrays. The comparison study of five AMPs (polyhemusin-I, P-Der, Lfcin B, PR-39, and Bac 7) showed only nine common protein targets in all the five AMPs, whereas a total of 39 and 43 common protein targets were identified among the two marine AMPs (polyphemusin-I and P-Der) and three terrestrial AMPs (Lfcin B, PR-39 and Bac7), respectively. To further reveal the target pattern of marine and terrestrial AMPs, the enrichment results obtained from common protein targets of marine AMPs with terrestrial AMPs were compared. The comparison result indicated that AMPs have unique mechanism of action among marine or terrestrial AMPs. Hence, in this study, we have not only identified the intracellular protein targets of polyphemusin-I, but also revealed the protein target differences between marine AMPs and terrestrial AMPs.

## 1. Introduction

As a priority to search for novel antibiotics against the increasing number of multi-drug resistance pathogens, the naturally produced antimicrobial peptides (AMPs) found in the body fluids and tissues of all organisms (i.e., prokaryotic to eukaryotic) are extensively studied [1,2]. AMPs are the key component of humoral defense immunity that provides rapid reactions to invading pathogens and also stimulate the host’s immune responses [3]. Inside the host body, AMPs inhibit the action of pathogenic cytokines, like lipoteichoic acid and lipopolysaccharide (LPS) [4]. AMPs are unique, short sequence peptides (<50 amino acids) with a wide range of antimicrobial activities (ranging from bacteria, fungi to other pathogens). Both physiochemical properties and mechanisms of action are related to the secondary structure and net charge of AMPs [5]. Mostly, cationic AMPs (with net positive charge at physiological pH) are of amphipathic nature that interacts with the negatively charged phospholipids of bacterial membranes. This electrostatic interaction is considered as one of the potential and well-recognized mechanisms of action of AMPs that can disrupt bacterial cell membranes causing leakage of ions, metabolites, and others [2,6]. Besides membrane disruption, AMPs are reported to penetrate or translocate across the membrane providing localization of AMP to the cytoplasm where they target cytoplasmic molecules [7,8,9,10]. Further studies have depicted several intracellular (i.e., cytoplasmic) targets of AMPs, such as nucleic acid (DNA/RNA), protein, enzyme, and others [11,12]. Notably, it is suggested that unlike single target antibiotics, AMPs have multiple targets exerting multi-targets mechanism of actions [6,13].

Polyphemusin-I is a marine AMP obtained from hemocyte debris of *Lumulus polyphemus* (American horse-shoe crab) [14]. Polyphemusin-I, an 18 amino acid residues AMP, has an antiparallel β-hairpin structure [15] and a net positive charge of +7 with hydrophobicity and amphipathic moments of −0.56 and 0.48, respectively [16]. Polyphemusin-I exerts high potential antimicrobial activities against a wide range of pathogens [14] with reported minimum inhibitory concentrations (MICs) against *Escherichia coli* and *Candida albicans* of 0.5 µg/mL and 1.0 µg/mL, respectively [16].

While initial observation revealed the interaction of polyphemusin-I with bacterial LPS [14], subsequent studies have shown the translocation of polyphemusin-I across model membranes [13,15,16] as well as *Escherichia coli* cytoplasm [17]. Moreover, at a concentration lower than the MIC, polyphemusin-I permeabilizes the bacterial membrane and gains access to the cytoplasm [16]. Despite the evidence of internalization of polyphenusin-I to *Escherichia coli* cytoplasm, its intracellular targets are not yet reported. In this study, we have used the powerful high-throughput *Escherichia coli* proteome microarrays [18,19], containing the entire proteome of *Escherichia coli* K12, for the systematic and comprehensive identification of the entire protein targets range of polyphemusin-I. The knowledge of the entire targets will aid in understanding the detail mechanisms of action of polyphemusin-I. The protein targets of polyphemusin-I were identified statistically from the quadruplicate *Escherichia coli* proteome chip assays of polyphemusin-I. These identified protein targets of polyphemusin-I were subjected to different bioinformatics analysis to understand the potential antibacterial mechanism of polyphemusin-I. Furthermore, we compared the identified protein targets of polyphemusin-I with the previously identified protein targets of other AMPs (P-Der, Lfcin B, PR-39, and Bac 7) using *Escherichia coli* proteome chips [20,21,22], to understand the target pattern difference between marine (polyphemusin-I and P-Der) and terrestrial AMPs (Lfcin B, PR-39, and Bac 7).

## 2. Results

### 2.1. Escherichia coli Proteome Microarrays Assays of Polyphemusin-I

**Figure 1:**
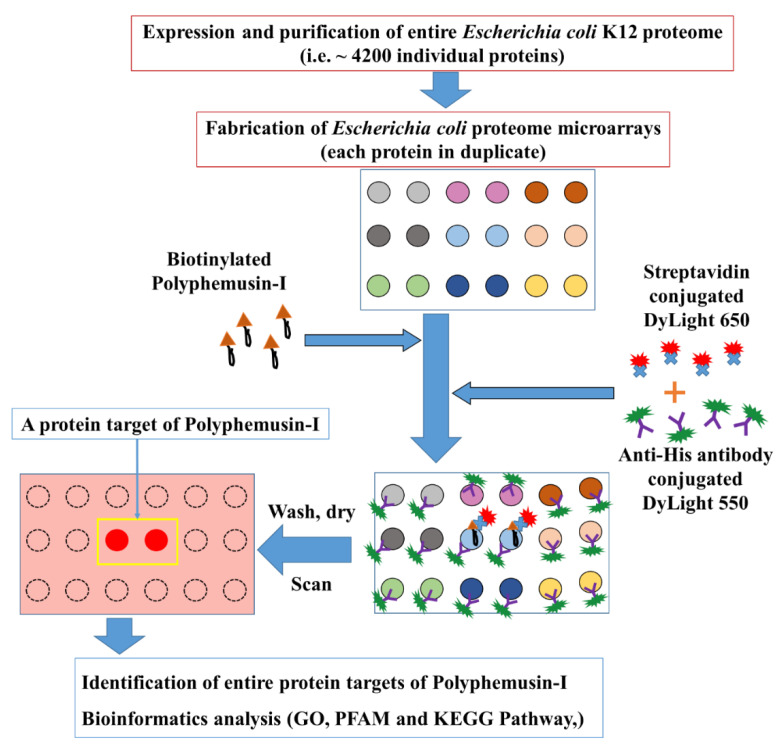
Schematic diagram for the identification of the protein targets of polyphemusin-I using *Escherichia coli* proteome microarrays. The entire proteome of *Escherichia coli* K12 was individually purified and spotted in duplicate on aldehyde coated glass slides (different colors represent different proteins in duplicate) to fabricate *Escherichia coli* proteome microarrays. To screen and identify the protein targets, biotinylated Polyphemusin-I was probed on *Escherichia coli* proteome microarrays followed by probing with streptavidin conjugated DyLight 650 and anti-His antibody conjugated DyLight 550. Here, streptavidin conjugated DyLight 650 and anti-His antibody conjugated DyLight 550 facilitated the detection of biotinylated polyphemusin-I and 6×His tag *Escherichia coli* proteins on *Escherichia coli* proteome microarrays, respectively. Statistical cutoff parameters were used to identify the potential protein targets of polyphemusin-I and these identified protein targets were analyzed with bioinformatics tools, like gene ontology (GO), protein family (PFAM), and Kyoto Encyclopedia of Genes and Genomes (KEGG).

Biotinylated polyphemusin-I was probed on the high-throughput *Escherichia coli* proteome microarrays for systematical screening of the entire potential protein targets of polyphemusin-I. *Escherichia coli* proteome microarrays contained a total of ~4200 individually purified *Escherichia coli* K12 which were printed in duplicate spots of each protein on the aldehyde coated glass slides. The overall schematic diagram of this ongoing study is displayed in Figure 1, where the fabricated *Escherichia coli* proteome microarrays were employed for comprehensive identification of the entire protein targets of polyphemusin-I. The different colors of spots (in duplicate) represent individual *Escherichia coli* K12 protein (Figure 1). The protein targets of polyphemusin-I were statistically identified and subjected to bioinformatics analysis to discover the significant over-representation of proteins belonging to different functional groups like gene ontology (GO), Protein families (PFAM), and Kyoto Encyclopedia of Genes and Genomes (KEGG) pathways databases.

For *Escherichia coli* proteome microarrays assays of polyphemusin-I, the *Escherichia coli* proteome microarrays were firstly blocked with bovine serum albumin (BSA), then probed with biotinylated polyphemusin-I, followed by probing of streptavidin-conjugated DyLight 650 and anti-His antibody conjugated DyLight 550. Streptavidin-biotin detection system was applied for the detection of biotinylated polyphemusin-I interacting to its specific *Escherichia coli* proteins with streptavidin conjugated DyLight 650. Anti-His antibody conjugated DyLight 550 signal represented the quantitative amount of individually purified *Escherichia coli* proteins containing 6×His tag on the *Escherichia coli* proteome microarrays. These *Escherichia coli* proteome microarrays were scanned with two colors (red and green). The red spot signal represented the interaction of streptavidin conjugated DyLight 650 to biotinylated polyphemusin-I bound to specific *Escherichia coli* protein on *Escherichia coli* proteome microarrays. Whereas, the green spot signal represented the interaction of anti-His antibody conjugated DyLight 550 to 6×His tag of *Escherichia coli* proteins. For simplicity, the green signal of anti-His antibody conjugated DyLight 550 is not shown. As depicted in Figure 1, the duplicate spots (in red color) represent a protein target of polyphemusin-I on a single *Escherichia coli* proteome microarray. To insure reproducibility, a total of quadruplicate *Escherichia coli* proteome microarrays assays of polyphemusin-I were conducted.

### 2.2. Statistical Analysis of Escherichia coli Proteome Microarrays Data for the Identification of Potential Protein Targets of Polyphemusin-I

**Figure 2:**
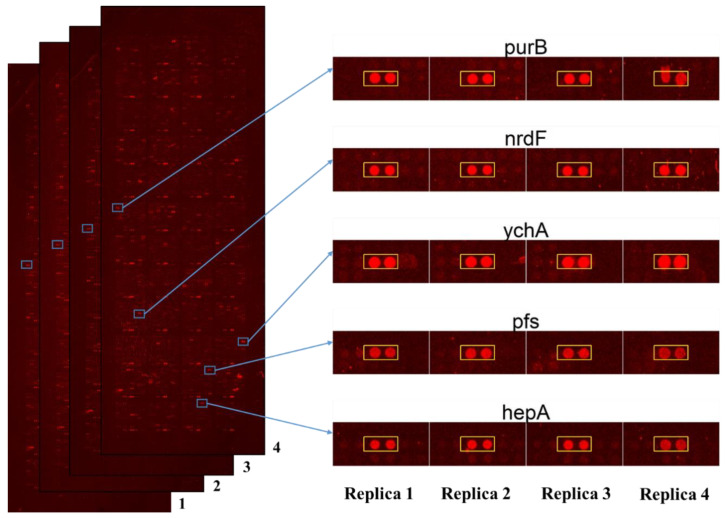
Images of *Escherichia coli* proteome microarrays and the representative protein targets of polyphemusin-I. The scan images of quadruplicate *Escherichia coli* proteome microarrays (numbered as 1, 2, 3 and 4) probed with polyphemusin-I (**left**). The enlarged images of representative protein targets of polyphemusin-I from quadruplicate *Escherichia coli* proteome microarrays (**right**). Each red spot (in duplicate) inside the square box represents the individual protein target of polyphemusin-I identified individually from four *Escherichia coli* proteome microarrays. Four replicated assays of *Escherichia coli* proteome microarrays with polyphemusin-I are represented by replicas 1, 2, 3 and 4, respectively.

The scanned results of quadruplicate *Escherichia coli* proteome microarrays of polyphemusin-I were individually opened with GenePix Pro and the data were exported as GenePix Result (GPR) file. Together, four GPR files from quadruplicate *Escherichia coli* proteome microarrays assays of polyphemusin-I were statistically analyzed. The statistical cutoff parameters included median scaling normalization, minimal intensity cutoff, coefficient of variable (CV) cutoff, two standard deviation cutoff (local cutoff of the intensity of signal higher than mean), 650/550 ratio cutoff, and finally eye validation of each protein candidate (in duplicate) in the quadruplicate *Escherichia coli* proteome microarrays, provided with 97 potential protein targets of polyphemusin-I. Detailed explanation of the criteria of protein targets identification of polyphemusin-I is mentioned in the materials and method section. The complete list of 97 protein targets of polyphemusin-I is displayed in Supplementary Table S1. Figure 2 displays the scanned images of *Escherichia coli* proteome microarrays assays of polyphemusin-I as well as the enlarged protein images (in duplicate) of representative protein targets of polyphemusin-I from the four independent *Escherichia coli* proteome microarrays assays of polyphemusin-I. The enlarged protein images (in duplicate) of the entire 97 protein targets of polyphemusin-I from the quadruplicate *Escherichia coli* proteome microarrays assays of polyphemusin-I are depicted in Supplementary Figure S1.

### 2.3. Enrichment Analysis in Gene Ontology of Protein Targets of Polyphemusin-I

The Universal Protein Resource (UniProt) database [23], the comprehensive resource of *Escherichia coli* K12, was explored to obtain Uniprot ID, protein names, updated protein ID, and cellular localization of the identified 97 protein targets of polyphemusin-I (Supplementary Table S1). As depicted in Supplementary Table S1, fifty-three of the total 97 identified protein targets of polyphemsuin-I were located inside the “cytoplasm”, whereas, three proteins were located on the “cell inner membrane”, one on the “plasma membrane”, one in the “periplasm” and rest 39 proteins had no specific location. The updated protein IDs of 97 protein targets of polyphemusin-I were used for enrichment analysis.

**Figure 3:**
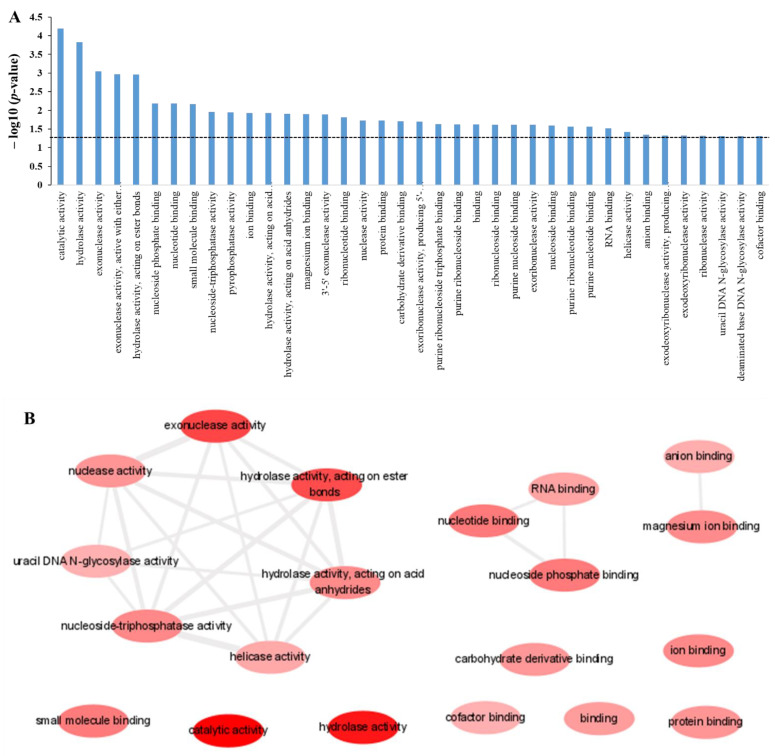
Enrichment in molecular function category of gene ontology (GO) for the protein targets of polyphemusin-I. (**A**) GO molecular function enriched terms analyzed by DAVID database using a *p*-value cutoff of ≤0.05 (dotted line represent *p*-value = 0.05). (**B**) REVIGO analysis for the selection of non-redundant GO term from the list of DAVID generated GO enrichment results in molecular function. Bubble indicated enriched non-redundant GO term and the color (red to pink) represent significant enrichment based on the *p*-value. The highly similar non-redundant GO terms are connected with the line where the width of the line represented the degrees of similarity.

To find the biological meaning and characterization of the identified protein targets of polyphemusin-I, GO enrichment analysis was performed by using the online Database for Annotation, Visualization and Integrated Discovery (DAVID) analysis platform [24]. In this study, all the three categories of GO enrichment analysis i.e., molecular function, biological process, and cellular component were analyzed. The GO enrichment results provided the over-represented of the protein targets of polyphemusin-I in specific GO term annotation. Moreover, enrichment in GO molecular function category provided the actions of protein at the molecular level of an organism. The GO molecular function enrichment analysis of the protein targets of polyphemusin-I using a *p*-value cutoff of ≤0.05, provided a list of 38 significantly enriched GO terms. The entire significant enriched GO terms of GO molecular function with their associated *p*-value (represented in –log10(*p*-value)) are depicted in Figure 3A. To show significant enrichment of each identified GO term a dotted line representing a *p*-value of 0.05 is drawn in Figure 3A. The largely unreadable list of 38 significantly enriched GO terms in GO molecular function categories might contain several redundant GO terms. Thus, to identify only the non-redundant GO terms enrichment in the list of GO enrichment terms in molecular function, Reduce and Visualized of Gene Ontology (REVIGO) were employed [25]. Table 1 shows the list of 38 enrichment GO terms in GO molecular function with detailed information on the identified protein targets in those categories, the corresponding *p*-value obtained from DAVID database, and the list of redundancy column indicating either the identified GO term is redundant or non-redundant from REVIGO. REVIGO analysis reduced the list of significant GO terms in GO molecular function from 38 to 20 and further provided the “interactive graph” image of 20 significantly enriched non-redundant GO terms where the highly similar GO terms were linked together (Figure 3B). The width of the connected line represented the degree of similarity between the two GO terms. As depicted in Figure 3B, the protein targets of polyphemusin-I were significantly enriched in “nuclease activity” and similar GO terms to nuclease activity (“exonuclease activity”, “hydrolase activity, acting on ester bonds”, “hydrolase activity, acting on acid anhydrides”, “helicase activity”, “nucleoside-triphosphatase activity” and “uracil DNA N-glycosylase activity”). Moreover, the protein targets of polyphemusin-I also showed enrichment in “RNA binding”, “nucleotide binding” and “nucleoside phosphate binding”. The identified protein targets of polyphemusin-I are ion and cofactor binding proteins, thus it was obvious to observe enrichment in “ion binding”, “anion binding”, “magnesium binding”, and “cofactor binding”. Analysis of other enriched GO terms also indicated enrichment of polyphemusin-I in nucleic acid (DNA and RNA)-associated molecular functions.

**Figure 4:**
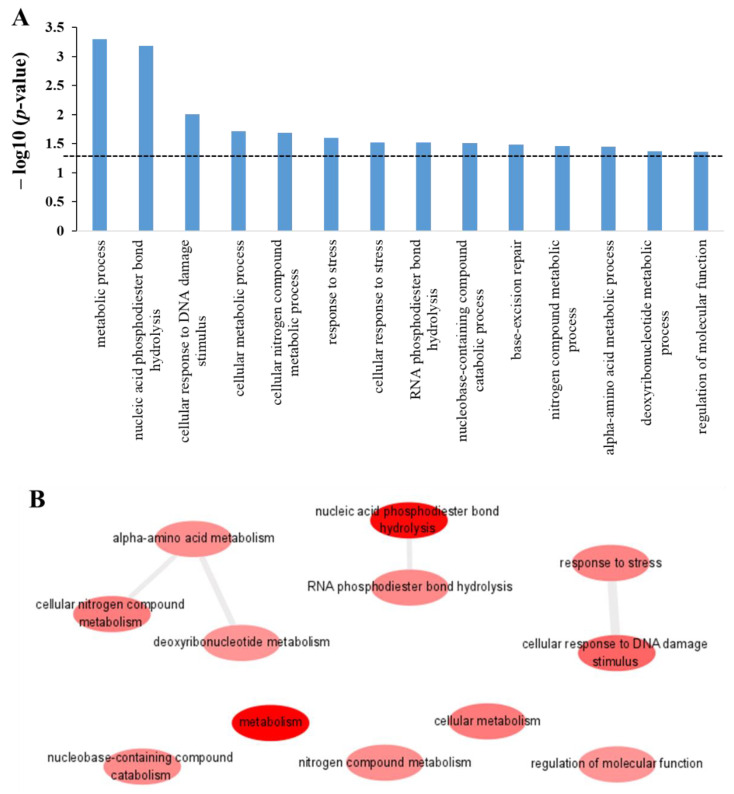
Enrichment in biological process category of gene ontology (GO) for the protein targets of polyphemusin-I. (**A**) GO biological process enriched terms analyzed by DAVID database and the use of *p*-value cutoff ≤0.05 to select significant enrichment (dotted line represent *p*-value = 0.05). (**B**) REVIGO analysis for the selection of non-redundant GO term from the list of DAVID generated GO enrichment results in biological process. Bubble indicated enriched non-redundant GO term and the color (red to pink) represent significant enrichment based on the *p*-value. The highly similar non-redundant GO terms showed the connection and the width of line represent the degree’s similarity.

GO biological process on the other hand execute information at the cellular or organism level with certain biological objectives accomplished by the combination of one or more molecular function(s). The GO enrichment analysis for the protein targets of polyphemusin-I in the biological process was performed using DAVID database. Figure 4A depicts the significant enrichment GO terms identified in biological process with their respective *p*-value (*p*-value cutoff of ≤0.05 was applied and the dotted line represents *p*-value = 0.05). In total 14 significantly enriched GO terms were identified in GO biological process. These GO terms were further analyzed by REVIGO and the results showed 12 GO terms out of 14, were non-redundant enrichment GO terms in biological process. The significantly enriched GO terms in biological process followed by the *p*-value, of the number of identified protein targets of polyphemusin-I, and the total proteins involved in these GO terms are displayed in Table 2. Moreover, REVIGO analyzed information on the redundancy of all the identified GO terms in GO biological process are mentioned in a separate column in Table 2. The 12 identified non-redundant GO terms in GO biological process showed similarity with a few GO terms which are connected by a line and the width of the line indicates the degree of similarity between these GO terms (Figure 4B). This similarity is based on performing a similar task or involvement in the same biological process (i.e., child-parent relationship of all the annotated GO terms). As displayed in Figure 4B, the protein targets of polyphemusin-I were significantly enriched in “alpha-amino acid metabolism”, “cellular nitrogen compound metabolism” and “deoxyribonucleotide metabolism”. Moreover, these three GO terms are related (Figure 4B) as the metabolism of alpha-amino acid are required for the metabolism of “cellular nitrogen compound” and “deoxyribonucleotide” molecules. Furthermore, the hydrolysis activity of phosphodiester bond in DNA and RNA are similar thus, the significantly enriched GO terms “nucleic acid phosphodiester bond hydrolysis” and “RNA phosphodiester bond hydrolysis” showed the connection between these two GO terms.

Similarly, the enriched GO term “cellular response to DNA damage stimulus” is a part of the enriched GO term of “response to stress”. Thus, these two GO terms show similarity, as depicted in Figure 4B. Moreover, other non-redundant GO terms enriched in GO biological process are “metabolism”, “cellular metabolism”, “nitrogen compound metabolism”, “nucleobase-containing compound catabolism” and “regulation of molecular function”. Taken together, most of the significantly enriched GO terms in GO biological process are related to nucleic acid (i.e., DNA and RNA) biological processes. Similar to the enrichment finding in molecular function, biological process also indicated nucleic acid associated proteins as the targets of polyphemusin-I.

**Figure 5:**
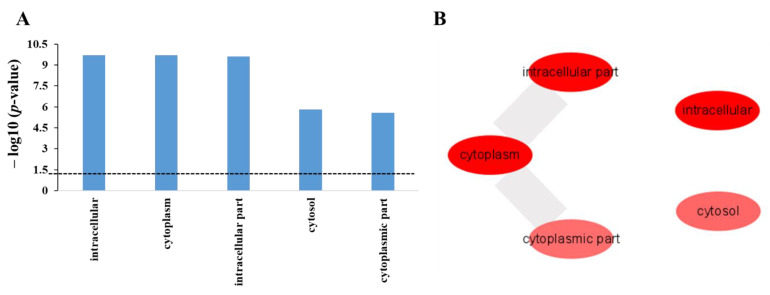
Enrichment in cellular component category of gene ontology (GO) for the protein targets of polyphemusin-I. (**A**) GO cellular component enriched term analyzed by DAVID database with *p*-value cutoff of ≤0.05 for the selection of significant enrichment terms (dotted line represent *p*-value = 0.05). (**B**) REVIGO analysis for the selection of non-redundant GO term from the list of DAVID generated GO enrichment results in cellular component. Bubble indicated enriched non-redundant GO term and the color (red to pink) represent significant enrichment based on *p*-value. The highly similar non-redundant GO term showed connection and the width of line represent degree’s similarity.

The third category of GO in cellular component provides the location of proteins inside the cell. Figure 5A shows the five significantly enriched GO terms in cellular component of GO and their corresponding p-value that were analyzed for the protein targets of polyphemusin-I using DAVID database. The *p*-value cutoff of ≤0.05 was applied for the selection of significantly enriched GO terms in cellular component of GO (in Figure 5A, dotted line indicates *p*-value = 0.05). These five highly significant enrichment GO terms— “intracellular”, “cytoplasm”, “intracellular part”, “cytosol” and “cytoplasmic part”—were subjected to REVIGO to identify the non-redundant GO term enrichment and also the similarity between the non-redundant GO terms. As depicted in Figure 5B, all the five GO terms from DAVID enrichment analysis in GO cellular component were non-redundant enrichment terms. A high degree of similarity indicated by a wide broader line was observed between enrichment terms of “cytoplasm”, “intracellular part”, and “cytoplasmic part”. This observation was expected as these were the intracellular protein targets of polyphemusin-I. Moreover, several soluble proteins are present in the intracellular fluid found inside cytoplasm and organelles, enrichment in GO term “cytosol” indicated the location of protein targets of polyphemusin-I in these fluids. The enrichment terms in GO cellular component are depicted in Table 3 with corresponding *p*-value, a total protein identified from protein targets of polyphemusin-I, total proteins in *Escherichia coli* belonging to the specific category, and the REVIGO results of showing redundancy of the identified GO enrichment term in cellular component.

### 2.4. Enrichment Analysis in Domain of the Protein Targets of Polyphemusin-I

In the protein families and subfamilies, the proteins are grouped based on their similarity in structure and function, hence, the protein families provide further insight into the phenotypic relation of proteins. Thus, to identify the biologically relevant protein targets of polyphemusin-I which share closely associated functions, Protein Families (PFAM) enrichment analysis using the DAVID database was performed. The significant enrichment in the protein domain of PFAM analysis with the applied *p*-value cutoff of ≤0.05 is shown in Table 4. These enrichment terms accord with the GO enrichment results, as significant enrichment is seen in RNA binding protein families (i.e., “S1 RNA binding domain” and “ribonucleotide reductase, small chain”) and DNA interacting protein families (i.e., “3′-5′ exonuclease” and “Uracil DNA glycosylase superfamily”). Other significantly enriched protein domains from the protein targets of polyphemusin-I, analyzed by PFAM, were “aminotransferase class I and II” and “uncharacterized protein family (UPF0149)”. Taken together, protein targets of polyphemusin-I showed significant domain enrichment in proteins binding to RNA and DNA. These results also indicated the associated proteins of DNA and RNA as the intracellular target of polyphemusin-I.

### 2.5. Enrichment Analysis in Pathway of Protein Targets of Polyphemusin-I

To analyze the biological pathways related to the identified protein targets of polyphemusin-I, the protein targets of polyphemusin-I were subjected to pathway enrichment analysis by KEGG database through the online analysis platform of DAVID database. Moreover, to obtain significant enrichment in pathways analysis, *p*-value cutoff of ≤0.05 was applied. Table 5 displays the pathway enrichment results of the protein targets of polyphemusin-I. The three enriched pathway terms were “base excision repair”, “purine metabolism” and “pyrimidine metabolism”. Metabolism of purine and pyrimidine indicates the key components required for the synthesis of nucleic acid (i.e., both DNA and RNA) whereas enrichment in “base excision repair”, represent the involvement of protein targets of polyphemusin in the DNA damage repair pathway. Pathway enrichment results also depicted the involvement of the protein targets of polyphemusin-I in DNA and RNA related pathways.

## 3. Discussion

In this study, we have explored the entire intracellular protein targets of polyphemsuin-I using *Escherichia coli* proteome microarrays. A previous study has shown that conjugation of biotin to polyphemusin-I (i.e., for biotinylated polyphemusin-I) did not affect the structure of polyphemusin-I, whereas a minimal effect on its antimicrobial activity [17], thus, biotinylated polyphemusin-I was used in this study. The systematic screening of polyphemusin-I using *Escherichia coli* proteome microarrays revealed 97 protein targets. These protein targets depicted enrichment in several categories of nucleic acid (DNA and RNA)-associated proteins. Previously, Hancock and his colleagues had predicted that polyphemusin-I might target DNA from their observation on polyphemusin-I localization study in *Escherichia coli* [17]. Our current study provided significant evidence that polyphemusin-I targets the proteins associated with DNA and RNA.

**Figure 6:**
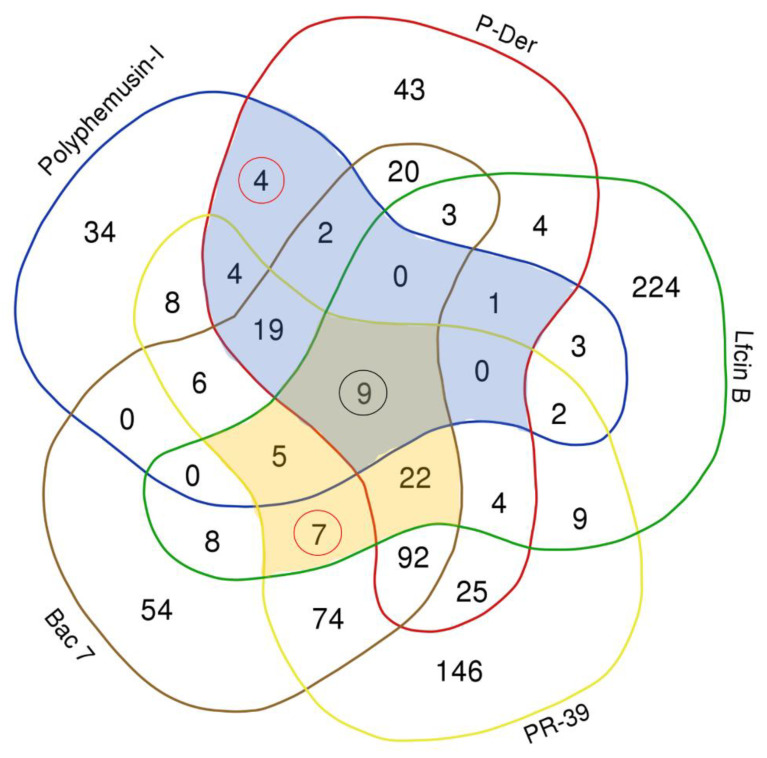
Venn diagram showing comparison of the protein targets of polyphemusin-I, P-Der, Lfcin B, PR-39 and Bac 7 identified using *Escherichia coli* proteome microarrays. The number of unique hits of polyphemusin-I, P-Der, LfcinB, PR-39 and Bac 7 were 34, 43, 224, 146, and 54, respectively. All the five AMPs (i.e., marine and terrestrial AMPs) shared nine protein targets (highlighted by black circle). The common protein targets between marine AMPs (polyphemusin-I, P-Der) were 39 proteins (shown under blue color region). Whereas 43 proteins (shown under yellow color region) were the common protein targets of terrestrial AMPs (Lfcin B, PR-39 and Bac7). The four and seven proteins (enclosed inside the circle) indicate the only common unique protein targets among marine AMPs and terrestrial AMPs, respectively.

To provide a comprehensive study, comparison between marine AMPs [i.e., polyphemusin-I (in this study) and hybrid of pleurocidin and dermaseptin (P-Der) (in the previous study)] and the previously studied terrestrial AMPs [i.e., lactoferricin B (Lfcin B), proline-arginine (PR)-rich AMPs (PR-39) and bactenecin 7 (Bac 7)] [20,21,22] were performed. Pleuricidin (sequence: ‘GWGSFF’-KKAAHVGKHVGKAALTHYL) is a marine AMP obtained from the skin of the winter flounder (*Pleuronectes americanus*) and dermaseptin (sequence: ‘ALWKTML’-KKLGTMALHAGKAALGAAADTISQTQ) is a terrestrial AMP isolated from the skin of the tarsier leaf frog (*Phyllomedusa tarsius*). P-Der (sequence: ALWKTML-KKAAHVGKHVGKAALTHYL-NH_2_) is synthesized by replacing the first six amino acids of pleurocidin with seven initial amino acids of dermaseptin. Hence, P-Der can be considered as a marine AMP. The total protein targets identified for polyphemusin-I, P-Der, Lfcin B, PR-39, and Bac 7 using *Escherichia coli* proteome microarrays were 97, 252, 301, 432, and 321, respectively. The Venn diagram, as depicted in Figure 6, showed the unique (i.e., present only in specific AMP) and common (overlap between different AMPs) protein targets among these five AMPs (i.e., polyphemusin-I, P-Der, Lfcin B, PR-39, and Bac 7). A total of nine common protein targets (highlighted by black color circle in Figure 6) were observed in all five AMPs. The unique protein targets of polyphemusin-I, P-Der, Lfcin B, PR-39, and Bac 7 were 34, 43, 224, 146, and 54 proteins, respectively. This comparison demonstrated that protein targets are mostly shared among the AMPs. As previously described, PR-39 and Bac 7 belong to the same AMP family (i.e., the cathelicidin AMPs family), thus they shared a total of 234 common protein targets [20]. Among the five AMPs, only four (rfbB, ybiU, tmcA, thrA) unique common protein targets were identified between marine AMPs (highlighted by circle in Figure 6), whereas, seven (aceK, mngB, glpK, yieH, ygeX, yphH and ybjI) unique common protein targets were identified of terrestrial AMPs (highlighted by circle in Figure 6).To obtain further insight between the shared protein targets of specific type (i.e., marine or terrestrial) AMPs, we looked for the shared protein targets only between marine AMPs or terrestrial AMPs. A total of 39 common protein targets were observed between marine AMPs (polyphemusin-I and P-Der) (blue color area in Figure 6). Among the three terrestrial AMPs (Lfcin B, PR-39, and Bac 7), only 43 common protein targets were identified (yellow color area in Figure 6).

Enrichment analyses using 39 common protein targets of marine AMPs (polyphemusin-I and P-Der) were performed to observe the common enrichment pattern between marine AMPs. Table 6 displayed the significant enrichment (with *p*-value cutoff of ≤0.05) of the common protein targets of marine AMPs in GO biological process, GO molecular function, KEGG pathway, and PFAM domain enrichment analysis. The common enrichment results of marine AMPs in biological process depicted significant enrichment in DNA related processes (“deoxyribonucleotide metabolic process”, “deoxyribonucleoside diphosphate metabolic process” and “negative regulation of sequence-specific DNA binding transcription factor activity”) and polyamine related processes (“polyamine metabolic process”, “polyamine biosynthetic process” and “spermidine biosynthesis process”). In molecular function, significant common enrichments of marine AMPs were observed for “hydrolase activity, acting on ester bond”, “hydrolase activity” and “catalytic activity”, which also indicate damage to DNA. Moreover, pathway enrichment results in “cysteine and methionine metabolism” also indicated targeting to DNA synthesis pathways. Enrichment in PFAM, also depicted targeting to DNA synthesis (i.e., “ribonucleotide reductase, small chain”). These analysis results demonstrated a common mechanism of marine AMPs in targeting DNA related proteins. Taken together, the common enrichment of marine AMPs, as well as unique enrichment of polyphemusin-I depicted nucleic acid associated proteins as the mechanism of action of polyphemusin-I.

**Figure 7:**
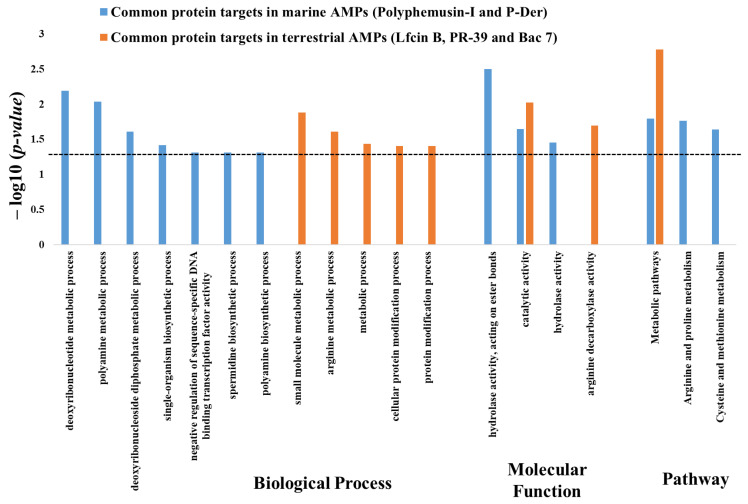
Comparison of enrichment terms obtained from the enrichment analysis of the common protein targets of marine AMPs (polyphemusin-I and P-Der) and terrestrial AMPs (Lfcin B, PR-39 and Bac 7). Enrichment analysis of common protein targets of marine AMPs (polyphemusin-I and P-Der) as well as for the common protein targets of terrestrial AMPs (Lfcin B, PR-39 and Bac 7) were performed, individually. The enrichment results were compared in GO biological process, molecular function and pathway. The *p*-value cutoff of ≤0.05 were applied to select the significant enrichment terms (dotted line represent *p*-value = 0.05).

Furthermore, enrichment analysis with the *p*-value cutoff of ≤0.05 were performed for the 43 common protein targets between the terrestrial AMPs (Lfcin B, PR-39, and Bac 7). To observe the difference in the target pattern, the enrichment results for the common protein targets of terrestrial AMPs (Lfcin B, PR-39, and Bac 7) were compared to the common enrichment between marine AMPs (polyphemusin-I and P-Der). As depicted in Figure 7, the comparison results mostly demonstrated unique enrichment between the common protein targets of marine AMPs and terrestrial AMPs, in GO biological process, GO molecular function, and pathway. The unique enrichment for the common protein targets of marine AMPs (polyphemusin-I and P-Der), as shown in Figure 7 and also mentioned in Table 6, pointed toward a targeting of DNA-associated proteins. On the other hand, the enrichment for the common protein targets of terrestrial AMPs (Lfcin B, PR-39, and Bac 7) showed significant enrichment in several metabolic related processes (Figure 7). Meanwhile, with nine common protein targets in all the five AMPs, two common enrichments were observed between the common targets of marine AMPs and terrestrial AMPs, i.e., one in molecular function (i.e., “catalytic activity”) and another in pathway (i.e., “metabolic pathways”).

In summary, marine organisms secrete potential AMPs that not only provide for their survival in the marine environment, but these AMPs are also potentially beneficial to mankind. To use these AMPs as therapeutical agents, a complete knowledge of their targets is a prerequisite. These targets provide basic and in-depth knowledge of the mechanism of action. Thus, in this study, the entire intracellular protein targets of polyphemusin-I were systematically identified using *Escherichia coli* proteome microarrays. Bioinformatics analysis of these protein targets provided significant enrichment in nucleic acid (DNA and RNA)-related processes, functions, and pathways. This finding provided the complete list of protein targets of polyphemusin-I as well as further indicated the mechanism of action of polyphemusin-I by targeting nucleic acids-associated proteins. Moreover, the comparison of polyphemusin-I with our previously identified AMPs (P-Der, Lfcin B, PR-39, and Bac 7) provided a better understanding between marine AMPs and terrestrial AMPs. Hence, the use of proteome microarrays has provided a robust platform for the identification of the entire protein targets of AMPs and we will keep on exploring the protein targets of other potential AMPs to broaden our knowledge and understanding of AMPs.

## 4. Materials and Methods

### 4.1. Expression and Purification of Entire Proteins of Escherichia coli K12 Strain

In this study, A complete Set of *Escherichia coli* K12 ORF Archive (ASKA) library [26] containing entire *Escherichia coli* K12 individual proteins i.e., ~4200 individual open reading frames (ORFs) constructed in plasmid pCA24N and transferred in *Escherichia coli* strain were used. Moreover, the entire proteins of *Escherichia coli* K12 were obtained using high-throughput expression and purification protocols adopted from a previous study [27].

For expression of entire *Escherichia coli* proteins, the ASKA library stock kept in a −80 °C freezer was thawed and cultured in 96-deep well plates containing 2× Luria-Bertani (LB) medium with 30 μg/mL of chloramphenicol incubated with shaking at 200 rpm at 37 °C for overnight. Overnight culture (8 µL) was transferred in new 96-deep well plates containing 800 µL 2× LB with 30 μg/mL chloramphenicol. The 96-deep well plates were incubated with shaking at 37 °C for ~4 h or till the optical density (OD_600nm_) value of the culture reached 0.4~0.7. For the protein expression, IPTG with the final concentration of 0.5 mM was then added into each well and further incubated with shaking at 37 °C for ~4 h. Finally, the *Escherichia coli* containing specific proteins were harvested by centrifugation for 5 min at 4000 rpm. The cell pellets were collected by discarding the supernatants and stored at −80 °C until the purification of proteins (usually less than 1 week).

For the high-throughput purification of entire *Escherichia coli* proteins, the cell pellets (stored at −80 °C) were re-suspended in 80 µL freshly prepared lysis buffer with several proteinase enzymes (CelLytic B cell lysis reagent, 50 mM NaH_2_PO_4_, 40 mM imidazole, 300 mM NaCl, 50 units/mL benzonase R Nuclease, 1 mg/mL lysozyme, 1 mM/mL phenylmethylsulfonyl fluoride (PMSF) and proteinase inhibitor cocktail). These mixtures were transferred into 96- well filter plates with pre-loaded Ni-NTA resins (bottom of filter plates were sealed) and the filter plates were incubated with shaking at 4 °C for 1.5 h to facilitate the binging between 6×His tag of *Escherichia coli* proteins and Ni-NTA resins. The filter plates with complexes of resin-protein were loaded to the reservoir plate by carefully removing the bottom cover. Each well of the filter plates was washed five times with wash buffer I at pH 8.0 (50 mM NaH_2_PO_4_, 30 mM imidazole, 300 mM NaCl, 10% glycerol, and 0.05% Triton X-100) followed by five further times washed with wash buffer II at pH 8.0 (50 mM NaH_2_PO_4_, 30 mM imidazole, 150 mM NaCl, 30% glycerol and 0.05% Triton X-100). Brief centrifugation was done to pass the unbound contents in the filter plates. The *Escherichia coli* protein in each well was eluted firstly with 25 μL elution buffer I at pH 7.5 (500 mM imidazole, 50 mM NaH_2_PO_4_, 150 mM NaCl, 30% glycerol, and 0.05% Triton X-100) followed by incubation with shaking at 4 °C for 30 min and collection of purified protein in 96-well reservoir plates by centrifugation at 1000 rpm for 30 seconds. Secondly, the proteins were eluted twice with 25 μL of elution buffer II at pH 7.5 (300 mM imidazole, 50 mM NaH_2_PO_4_, 150 mM NaCl, 30% glycerol, and 0.05% Triton X-100) followed by incubation with shaking at 4 °C for 30 min and collection of the purified protein by centrifugation at 1000 rpm for 30 seconds. A total of 75 μL eluted protein in each well of 96-well plate from ~4200 colonies were aliquot and store in −80°C for further use. Chemicals were purchased from Sigma Aldrich (Sigma Aldrich, St. Louis, MO, USA).

### 4.2. Fabrication of Escherichia coli Proteome Microarrays from the Individually Purified Entire Escherichia coli Proteome

The purified proteins of *Escherichia coli* K12 (expressed and purified using above mentioned protocols) were transferred from 96- well plates to 384 well plates using Liquidator 96 manual pipetting system (Mettler Toledo Rainin LLC, Oakland, CA, USA). SmartArrayer^TM^136 (CapitalBio Corp., Beijing, China), containing 48 microarrays spotter pins, a rinse tank, dry vacuum chamber, sonication tank, and a platform to load 136 glass slides were placed in a cold room at 4 °C, was used for fabrication of *Escherichia coli* proteome microarrays. Using the contact technique of SmartArrayer^TM^136, duplicate spots of each protein were printed on the aldehyde coated glass slide in 48 blocks with 48 microarrays spotter pins. The single uptake of a sample by one microarray pin facilitated spotting of around 300 uniform protein spots. To avoid contamination between the proteins, an optimized wash protocol was used for washing the 48 microarrays spotter pins after each duplicate printing. Moreover, optimized concentrations of positive controls and landmark proteins were printed in each block to assist in array alignment of the *Escherichia coli* proteome microarrays. Of the 136 glass slides used, 100 aldehyde-coated glass slides were for the fabrication of *Escherichia coli* proteome microarrays whereas the remaining 36 normal glass slides without any coating were used as pre-spotting slides that help in the removal of extra proteins solution on the outer surface of the microarrays printing pins. The temperature (4 °C) and humidity (<40%) of the cold room were strictly maintained to obtain a uniform spot of proteins on 100 aldehyde-coated glass slides. By following all the above steps, a batch of one hundred *Escherichia coli* proteome microarrays chips was fabricated. For immobilization of proteins on aldehyde glass slides (i.e., to facilitate covalent bonding between amine groups of protein and aldehyde coat on glass slides), the fabricated *Escherichia coli* proteome microarrays were left at 4 °C for overnight. Later, *Escherichia coli* proteome microarrays were placed in chip boxes, vacuum sealed, and store at −80 °C. The quality of shape, size, and uniformity for each protein spot on *Escherichia coli* proteome microarray was evaluated by probing with anti-His antibody-DyLight 550 (Rockland Immunochemicals Inc., Pottstown, PA, USA), washed, dry and scanned with LuxScan (10K Microarray Scanner; CapitalBio Corp., Beijing, China).

### 4.3. Escherichia coli Proteome Microarrays Assay for Polyphemusin-I

N-terminal biotinylated polyphemusin-I (RRWCFRVCYRGFCYRKCR–Biotin) was purchased (Kelowna International Scientific Inc., Taipei, Taiwan) and stored in aliquot at −80 °C. For *Escherichia coli* proteome microarrays assays with polyphemusin-I, *Escherichia coli* proteome microarrays stored in −80 °C were immersed in 1× PBS-T (0.05% Tween 20) and incubated at room temperature (RT) with 40 rpm of shaking for 2 min, to remove the non-immobilized proteins on *Escherichia coli* proteome microarrays. Then, *Escherichia coli* proteome microarrays were blocked with 3% bovine serum albumin (BSA; Sigma-Aldrich, St. Louis, MO, USA) in 1× PBS by incubating at RT with 40 rpm of shaking for 1 h. To remove excess BSA, the *Escherichia coli* proteome microarrays were washed once with 1× PBS-T (0.05% Tween 20) by incubating at RT with 40 rpm of shaking for 5 min. Then, *Escherichia coli* proteome microarrays were probed with 10 µM of biotinylated polyphemusin-I diluted in 1% BSA in 1× PBS. To facilitate binding between polyphemsin-I and *Escherichia coli* proteins, *Escherichia coli* proteome microarrays with biotinylated polyphemusin-I were incubated at RT with 40 rpm of shaking for 1 h. The unbound polyphemusin-I from *Escherichia coli* proteome microarrays were removed by washing with 1×PBS-T and incubating at RT with 40 rpm of shaking for 5 min, a total of three times. After washes, *Escherichia coli* proteome microarrays were further probed with streptavidin conjugated DyLight™ 650 and anti-His antibody conjugated DyLight™ 550 (Thermo Fisher Scientific, Waltham, MA, USA) and incubated at RT with 40 rpm of shaking for 1 h. *Escherichia coli* proteome microarrays were washed with 1× PBS-T by incubating at RT with 40 rpm of shaking for 5 min, for three times. To remove the 1× PBS-T, *Escherichia coli* proteome microarrays were centrifuged at 1000 rpm for one minute. Finally, the dried *Escherichia coli* proteome microarrays were scanned with LuxScan.

### 4.4. Identification of Protein Targets of Polyphemusin-I from Escherichia coli Proteome Microarrays

The scanned images of *Escherichia coli* proteome microarrays probed with polyphemusin-I from LuxScan were saved as TIF files. These TIF files of polyphemusin-I probed *Escherichia coli* proteome microarrays were opened with GenePix Pro 6.0 software (Axon Instruments, Union City, CA, USA) using two specific wavelengths of 650 and 550 nm. The name and location of each *Escherichia coli* protein on *Escherichia coli* proteome microarrays was saved in a file with GAL file format. To identify each protein spot on *Escherichia coli* proteome microarrays, the GAL file was opened in GenePix Pro 6.0. The protein spots on *Escherichia coli* proteome microarrays probed with polyphemusin-I were aligned for the identification of names of the *Escherichia coli* proteins bound to polyphemusin-I. These data were exported and saved as GPR files.

The GPR files of polyphemusin-I probed on *Escherichia coli* proteome microarrays were individually opened in excel. The data from all four repeats were copied and pasted into a single excel file. The quadruplicate data of *Escherichia coli* proteome microarrays probed with polyphemusin-I were analyzed together by applying several cutoff parameters to identify the most potential protein targets of polyphemusin-I. Firstly, the individual protein signal from quadruplicate proteome microarrays assays were normalized using median scaling normalization, independently for red signal (i.e., 650) representing binding signal of polyphemusin-I to *Escherichia coli* proteins and green signal representing *Escherichia coli* protein quantities. Secondly, the intensity of the binding signal between polyphemusin-I and *Escherichia coli* protein of each protein spot on *Escherichia coli* proteome microarrays were selected to be greater than 100. Thirdly, to obtain the more realistic result, the variation between the duplicate protein spots of individual *Escherichia coli* proteins on the individual *Escherichia coli* proteome microarrays were cutoff using coefficient variation (CV) lower than 0.5. After these cutoffs were applied, two other cutoff parameters were set to define the positive target of polyphemusin-I. Firstly, the intensity of each protein should be higher than the local cut-off, defined by two standard deviations (SD) above the signal mean for each spot. Secondly, the fold change intensity ratio of polyphemusin-I to anti-His antibody should be greater than 0.5. The generated list of potential protein targets of polyphemusin-I was validated by the eyes to confirm the signal of potential protein targets on quadruplicate *Escherichia coli* proteome microarrays.

### 4.5. Bioinformatics Analysis

#### 4.5.1. Information on the *Escherichia coli* K12 Protein Targets of Polyphemusin-I

On 20 December 2020, UniProt database (https://www.uniprot.org/) was used to confirm and update information on *Escherichia coli* K12 protein names for all the identified *Escherichia coli* K12 protein targets of polyphemusin-I [23]. Venn diagram was generated using online platform (http://bioinformatics.psb.ugent.be/webtools/Venn/) on 21 February 2021.

#### 4.5.2. Enrichment Analysis of Protein Targets of Polyphemusin-I

To conduct the enrichment analysis of the protein targets of polyphemusin-I, DAVID database version 6.8 (https://david.ncifcrf.gov/) was employed on 23 December 2020 [24]. The DAVID database provides an enrichment analysis platform for GO, KEGG, and PFAM. For this enrichment analysis, *Escherichia coli* strain K-12 sub-strain MG1655 was used as selected species for background parameter and official gene symbol as selection identifier on the online analysis platform of DAVID. GO provided enrichment analysis results in three categories (molecular function, biological process, and cellular component). Moreover, GO enrichment results of protein targets of polyphemusin-I in all the categories were obtained using ALL-GO-level. KEGG enrichment analysis provided enrichment on pathways whereas PFAM provided domain enrichment with the frequency of the protein targets of polyphemusin-I in certain annotation terms. A stringent cutoff of *p*-value ≤ 0.05 was applied in all analysis tools for the selection of highly significant enrichment terms in each category. All the obtained enrichment results were further analyzed in excel.

#### 4.5.3. Re-Enrichment Analysis for Non-Redundant GO Term from the GO Enrichment Analysis Results

The significant enrichment results of protein targets of polyphemusin-I in GO categories (from DAVID database) were further analyzed using REVIGO database (http://revigo.irb.hr/) to obtain only non-redundant enrichment GO terms [25], on 27 December 2020. For this analysis, the list of GO ID followed by *p*-value was input and the default parameter with “allowed similarity” of medium (0.7) was selected.

## Figures and Tables

**Table 1:** Enrichment analysis of protein targets of polyphemusin-I in molecular function category of Gene Ontology (GO).

Term_ID	Enrichment in GO Molecular Function	*p*-Value	Hit in This Category	Total Gene in This Category	Redundancy
GO:0000287	Magnesium ion binding	0.012486	11	178	Non-redundant
GO:0003824	Catalytic activity	0.0000646	68	2036	Non-redundant
GO:0004527	Exonuclease activity	0.000896	6	31	Non-redundant
GO:0016796	Exonuclease activity, active with either ribo- or deoxyribonucleic acids and producing 5′-phosphomonoesters	0.001072	5	19	Redundant
GO:0004540	Ribonuclease activity	0.048466	4	33	Redundant
GO:0004532	Exoribonuclease activity	0.024589	3	10	Redundant
GO:0004529	Exodeoxyribonuclease activity	0.04661	3	14	Redundant
GO:0016895	Exodeoxyribonuclease activity, producing 5′-phosphomonoesters	0.04661	3	14	Redundant
GO:0016896	Exoribonuclease activity, producing 5′-phosphomonoesters	0.019993	3	9	Redundant
GO:0008408	3′-5′ exonuclease activity	0.012774	4	20	Redundant
GO:0005488	Binding	0.023957	66	2262	Non-redundant
GO:0000166	Nucleotide binding	0.006481	28	681	Non-redundant
GO:0017076	Purine nucleotide binding	0.02744	20	484	Redundant
GO:0035639	Purine ribonucleoside triphosphate binding	0.023405	20	476	Redundant
GO:0001883	Purine nucleoside binding	0.024367	20	478	Redundant
GO:0001882	Nucleoside binding	0.025361	20	480	Redundant
GO:0032550	Purine ribonucleoside binding	0.023882	20	477	Redundant
GO:0032549	Ribonucleoside binding	0.024367	20	478	Redundant
GO:0032555	Purine ribonucleotide binding	0.026909	20	483	Redundant
GO:0032553	Ribonucleotide binding	0.015182	22	521	Redundant
GO:0048037	Cofactor binding	0.049752	13	285	Non-redundant
GO:0016787	Hydrolase activity	0.000148	33	694	Non-redundant
GO:0097367	Carbohydrate derivative binding	0.019759	22	534	Non-redundant
GO:0036094	Small molecule binding	0.006868	29	718	Non-redundant
GO:0043167	Ion binding	0.011802	32	852	Non-redundant
GO:0005515	Protein binding	0.018817	33	916	Non-redundant
GO:0004844	Uracil DNA N-glycosylase activity	0.049597	2	2	Non-redundant
GO:0097506	Deaminated base DNA N-glycosylase activity	0.049597	2	2	Redundant
GO:0003723	RNA binding	0.03047	10	176	Non-redundant
GO:0043168	Anion binding	0.044748	7	104	Non-redundant
GO:0004386	Helicase activity	0.038039	4	30	Non-redundant
GO:1901265	Nucleoside phosphate binding	0.006481	28	681	Non-redundant
GO:0016788	Hydrolase activity, acting on ester bonds	0.001108	15	224	Non-redundant
GO:0016817	Hydrolase activity, acting on acid anhydrides	0.012267	14	262	Non-redundant
GO:0004518	Nuclease activity	0.01875	7	85	Non-redundant
GO:0017111	Nucleoside-triphosphatase activity	0.011068	13	230	Non-redundant
GO:0016462	Pyrophosphatase activity	0.011191	14	259	Redundant
GO:0016818	Hydrolase activity, acting on acid anhydrides, in phosphorus-containing anhydrides	0.0119	14	261	Redundant

**Table 2:** Enrichment analysis of protein targets of polyphemusin-I in GO of biological process category.

Term_ID	Enrichment in GO Biological Process	*p*-Value	Hit in This Category	Total Gene in This Category	Redundancy
GO:0008152	metabolic process	0.000502	65	2321	Non-redundant
GO:0065009	regulation of molecular function	0.043287	5	60	Non-redundant
GO:0090305	nucleic acid phosphodiester bond hydrolysis	0.00065	7	49	Non-redundant
GO:0006974	cellular response to DNA damage stimulus	0.009881	13	256	Non-redundant
GO:0033554	cellular response to stress	0.029973	14	333	Redundant
GO:0006284	base-excision repair	0.03258	3	13	Redundant
GO:0044237	cellular metabolic process	0.019431	57	2126	Non-redundant
GO:0006807	nitrogen compound metabolic process	0.034615	43	1519	Non-redundant
GO:1901605	alpha-amino acid metabolic process	0.035589	9	172	Non-redundant
GO:0034655	nucleobase-containing compound catabolic process	0.030997	5	54	Non-redundant
GO:0034641	cellular nitrogen compound metabolic process	0.020584	38	1258	Non-redundant
GO:0090501	RNA phosphodiester bond hydrolysis	0.030508	4	31	Non-redundant
GO:0009262	deoxyribonucleotide metabolic process	0.042623	3	15	Non-redundant
GO:0006950	response to stress	0.025047	19	505	Non-redundant

**Table 3:** Enrichment analysis of protein targets of polyphemusin-I in GO of cellular component category.

Term_ID	Enrichment in GO Cellular Component	*p*-Value	Hit in This Category	Total Gene in This Category	Redundancy
GO:0005622	Intracellular	1.94 × 10^−10^	54	1613	Non-redundant
GO:0005737	Cytoplasm	2.04 × 10^−10^	52	1471	Non-redundant
GO:0044424	Intracellular part	2.41 × 10^−10^	53	1546	Non-redundant
GO:0005829	Cytosol	1.52 × 10^−6^	39	1049	Non-redundant
GO:0044444	Cytoplasmic part	2.61 × 10^−6^	39	1069	Non-redundant

**Table 4:** Enrichment categories in protein families (PFAM) domain for the protein targets of polyphemusin-I.

ID	Enrichment in PFAM	*p*-Value	Hit in This Category	Total Gene in This Category	Protein Targets in This Category
PF00575	S1 RNA binding domain	0.00058	4	8	NUSA, RNR, RPSA, RNG
PF00155	Aminotransferase class I and II	0.033687	3	13	MALY, KBL, ALAA
PF00268	Ribonucleotide reductase, small chain	0.044827	2	2	NRDF, NRDB
PF03695	Uncharacterized protein family (UPF0149)	0.044827	2	2	YGFB, YECA
PF01612	3′-5′ exonuclease	0.044827	2	2	POLA, RND
PF03167	Uracil DNA glycosylase superfamily	0.044827	2	2	MUG, UNG

**Table 5:** Enrichment categories in Kyoto Encyclopedia of Genes and Genomes (KEGG) pathways for the protein targets of Polyphemusin-I.

ID	Enrichment in KEGG Pathway	*p*-Value	Hit in This Category	Total Gene in This Category	Protein Targets in This Category
eco03410	Base excision repair	0.037412	3	14	POLA, MUG, UNG
eco00230	Purine metabolism	0.04109	6	87	PURB, ADD, POLA, NRDF, MAZG, NRDB
eco00240	Pyrimidine metabolism	0.046694	5	62	POLA, NRDF, MAZG, NRDB, PSUK

**Table 6:** Enrichment results for the 39 common protein targets of polyphemusin-I and P-Der.

Term_ID	Enrichment in Biological Process	*p*-Value	Hit in This Category	Total Gene in This Category
GO:0009262	Deoxyribonucleotide metabolic process	0.0064	3	15
GO:0006595	Polyamine metabolic process	0.0092	3	18
GO:0009186	Deoxyribonucleoside diphosphate metabolic process	0.0245	2	3
GO:0044711	Single-organism biosynthetic process	0.0384	10	584
GO:0043433	Negative regulation of sequence-specific DNA binding transcription factor activity	0.0485	2	6
GO:0008295	Spermidine biosynthetic process	0.0485	2	6
GO:0006596	Polyamine biosynthetic process	0.0485	2	6
	**Enrichment in biological process**			
GO:0016788	Hydrolase activity, acting on ester bonds	0.0032	8	224
GO:0003824	Catalytic activity	0.0226	25	2036
GO:0016787	Hydrolase activity	0.0349	12	694
	**Enrichment in pathway**			
eco01100	Metabolic pathways	0.0160	11	685
eco00330	Arginine and proline metabolism	0.0172	3	25
eco00270	Cysteine and methionine metabolism	0.0228	3	29
	**Enrichment in PFAM**			
PF00268	Ribonucleotide reductase, small chain	0.0168	2	2
PF02613	Nitrate reductase delta subunit	0.0415	2	5
PF00258	Flavodoxin	0.0496	2	6

## Data Availability

Not applicable.

## Supplementary Materials

The following are available online at https://www.mdpi.com/article/10.3390/ijms22179158/s1.
