# Supplementary material for: Systematical Screening of Intracellular Protein Targets of Polyphemusin-I Using Escherichia coli Proteome Microarrays
Source: Int J Mol Sci. 2021 Aug 25;22(17):9158. doi: 10.3390/ijms22179158 (PMC8431070; doi:10.3390/ijms22179158)
Supplement: Supplementary file 1 [file ijms-22-09158-s001.zip › ijms-1301746-supplementary.pdf]

Article

Systematical Screening of Intracellular Protein Targets of Polyphemusin-I Using *Escherichia coli* Proteome Microarrays

Pramod Shah [1, 2] and Chien-Sheng Chen [1,3,*]

Supplementary Table S1. List of 97 protein targets of Polyphemusin-I identified from the quadruplicate *Escherichia coli* proteome microarrays. The protein ID, uniprot ID, protein names, updated protein ID and the cellular location of each protein in the *Escherichia coli* for the 97 protein targets of Polyphemusin-I are depicted.

Protein ID	Uniprot ID	Protein names	Updated ID	Cellular location
nrdF	P37146	Ribonucleoside-diphosphate reductase 2 subunit beta	nrdF	cytoplasm
hemL	P23893	Glutamate-1-semialdehyde 2,1-aminomutase	hemL	cytoplasm
ygfB	P0A8C4	UPF0149 protein YgfB	ygfB	Cytosol
nrdB	P69924	Ribonucleoside-diphosphate reductase 1 subunit beta	nrdB	cytoplasm; cytosol
speA	P21170	Biosynthetic arginine decarboxylase	speA	Periplasm
amyA	P26612	Cytoplasmic alpha-amylase	amyA	cytoplasm
ychA	P0AGM5	UPF0162 protein YchA	ychA	N/A
sseB	P0AFZ1	Protein SseB	sseB	N/A
polA	P00582	DNA polymerase I	polA	cytoplasm
yggD	P11663	Fumarase E	fumE	N/A
yqiA	P0A8Z7	Esterase YqiA	yqiA	N/A
ygjR	P42599	Uncharacterized oxidoreductase YgjR	ygjR	N/A
yciU	P0A8L7	UPF0263 protein YciU	yciU	N/A
purB	P0AB89	Adenylosuccinate lyase	purB	Cytosol
yliJ	P0ACA7	Glutathione S-transferase GstB	gstB	cytosol; cytoplasm
rho	P0AG30	Transcription termination factor Rho	rho	Cytosol; membrane
yegK	P76395	Serine/threonine-protein phosphatase 3	pphC	N/A
ypfI	P76562	tRNA(Met) cytidine acetyltransferase TmcA	tmcA	cytoplasm
yeeI	P76346	Protein MtfA	mtfA	cytoplasm
rsxC	P77611	Ion-translocating oxidoreductase complex subunit C	rsxC	Cell inner membrane
yegV	P76419	Uncharacterized sugar kinase YegV	yegV	N/A
ydhS	P77148	Uncharacterized protein YdhS	ydhS	N/A
fliH	P31068	Flagellar assembly protein FliH	fliH	Cytoplasm
yjiA	P24203	P-loop guanosine triphosphatase YjiA	yjiA	cytosol; cytoplasm
yfcM	P76938	Elongation factor P hydroxylase	epmC	N/A
rpoN	P24255	RNA polymerase sigma-54 factor	rpoN	protein-DNA complex
hslO	P0A6Y5	33 kDa chaperonin	hslO	Cytoplasm
sbcB	P04995	Exodeoxyribonuclease I	sbcB	N/A

yihI	P0A8H6	Der GTPase-activating protein YihI	yihI	cytosol
yrfG	P64636	GMP/IMP nucleotidase YrfG	yrfG	cytosol
yjgD	P0AF90	Regulator of ribonuclease activity B	rraB	cytoplasm
ftsK	P46889	DNA translocase FtsK	ftsK	Cell inner membrane
yihX	P0A8Y3	Alpha-D-glucose 1-phosphate phosphatase YihX	yihX	N/A
hepA	P60240	RNA polymerase-associated protein RapA	rapA	cytoplasm
parE	P20083	DNA topoisomerase 4 subunit B	parE	cytosol
tauD	P37610	Alpha-ketoglutarate-dependent taurine dioxygenase	tauD	cytosol; cytoplasm
rpsA	P0AG67	30S ribosomal protein S1	rpsA	cytoplasm
speE	P09158	Polyamine aminopropyltransferase	speE	cytoplasm
yeiC	P30235	Pseudouridine kinase	psuK	N/A
dhaM	P37349	PEP-dependent dihydroxyacetone kinase, phosphoryl donor subunit DhaM	dhaM	cytosol
obgE	P42641	GTPase ObgE/CgtA	obgE	cytoplasm
ygjF	P0A9H1	G/U mismatch-specific DNA glycosylase	mug	cytoplasm
pfs	P0AF12	5'-methylthioadenosine/S-adenosylhomocysteine nucleosidase	mtnN	cytosol
yidJ	P31447	Uncharacterized sulfatase YidJ	yidJ	N/A
ygaP	P55734	Inner membrane protein YgaP	ygaP	Cell inner membrane
rng	P0A9J0	Ribonuclease G	rng	Cytoplasm; Cell inner membrane
yecA	P0AD05	Uncharacterized protein YecA	yecA	N/A
ybeX	P0AE78	Magnesium and cobalt efflux protein CorC	corC	Plasma Membrane
gadB	P69910	Glutamate decarboxylase beta	gadB	Cytoplasm ; Membrane
yggW	P52062	Heme chaperone HemW	hemW	cytoplasm
yjfI	P0AF76	Uncharacterized protein YjfI	yjfI	N/A
ung	P12295	Uracil-DNA glycosylase	ung	cytoplasm
pheT	P07395	Phenylalanine--tRNA ligase beta subunit	pheT	cytoplasm
ydfI	P77260	Uncharacterized oxidoreductase YdfI	ydfI	N/A
ycdY	P75915	Chaperone protein YcdY	ycdY	cytosol
puuA	P78061	amma-glutamylputrescine synthetase PuuA	puuA	N/A
ade	P31441	Adenine deaminase	ade	N/A
add	P22333	Adenosine deaminase	add	cytosol
yaeQ	P0AA97	Uncharacterized protein YaeQ	yaeQ	N/A
ydjL	P77539	Uncharacterized zinc-type alcohol dehydrogenase-like protein YdjL	ydjL	N/A
yegS	P76407	Lipid kinase YegS	yegS	cytoplasm
malY	P23256	Protein MalY	malY	N/A
ycfX	P75959	N-acetyl-D-glucosamine kinase	nagK	N/A
rfbB	P37759	dTDP-glucose 4,6-dehydratase 1	rfbB	cytosol
recE	P15032	Exodeoxyribonuclease 8	recE	N/A
hslU	P0A6H5	ATP-dependent protease ATPase subunit HslU	hslU	cytoplasm
yjjK	P0A9W3	Energy-dependent translational throttle protein EttA	ettA	cytoplasm

dmsD	P69853	Tat proofreading chaperone DmsD	dmsD	Cytoplasm ; Cell inner membrane
era	P06616	GTPase Era	era	Cytoplasm ; Cell inner membrane
eutJ	P77277	Ethanolamine utilization protein EutJ	eutJ	N/A
rnd	P09155	Ribonuclease D	rnd	cytoplasm
uxaB	P0A6L7	Altronate oxidoreductase	uxaB	cytosol
ybiU	P75791	Uncharacterized protein YbiU	ybiU	N/A
yfjX	P52139	Uncharacterized protein YfjX	yfjX	N/A
ptsI	P08839	Phosphoenolpyruvate-protein phosphotransferase	ptsI	cytoplasm
ispB	P0AD57	Octaprenyl diphosphate synthase	ispB	cytosol
hyaF	P19932	Hydrogenase-1 operon protein HyaF	hyaF	N/A
kbl	P0AB77	2-amino-3-ketobutyrate coenzyme A ligase	kbl	cytosol; cytoplasm
yghZ	Q46851	L-glyceraldehyde 3-phosphate reductase	gpr	N/A
mazG	P0AEY3	Nucleoside triphosphate pyrophosphohydrolase	mazG	N/A
ydiS	P77337	Probable electron transfer flavoprotein-quinone oxidoreductase YdiS	ydiS	N/A
fldA	P61949	Flavodoxin 1	fldA	cytosol; cytoplasm
fixA	P60566	Protein FixA	fixA	N/A
yqcA	P65367	Flavodoxin YqcA	yqcA	N/A
thrA	P00561	Bifunctional aspartokinase/homoserine dehydrogenase 1	thrA	N/A
yfbQ	P0A959	Glutamate-pyruvate aminotransferase AlaA	alaA	cytoplasm
recB	P08394	RecBCD enzyme subunit RecB	recB	cytosol
ydiQ	P76201	Putative electron transfer flavoprotein subunit YdiQ	ydiQ	N/A
nadC	P30011	Nicotinate-nucleotide pyrophosphorylase [carboxylating]	nadC	cytosol; cytoplasm
entF	P11454	Enterobactin synthase component F	entF	cytosol; Plasma Membrane
nusA	P0AFF6	Transcription termination/antitermination protein NusA	nusA	cytoplasm
yjgM	P39337	Uncharacterized N-acetyltransferase YjgM	yjgM	N/A
dos	P76129	Oxygen sensor protein DosP	dosP	N/A
ypfH	P76561	Esterase YpfH	ypfH	N/A
ybiT	P0A9U3	Probable ATP-binding protein YbiT	ybiT	N/A
rnr	P21499	Ribonuclease R	rnr	cytoplasm
fbp	P0A993	Fructose-1,6-bisphosphatase class 1	fbp	cytoplasm

Supplementary Figure S1

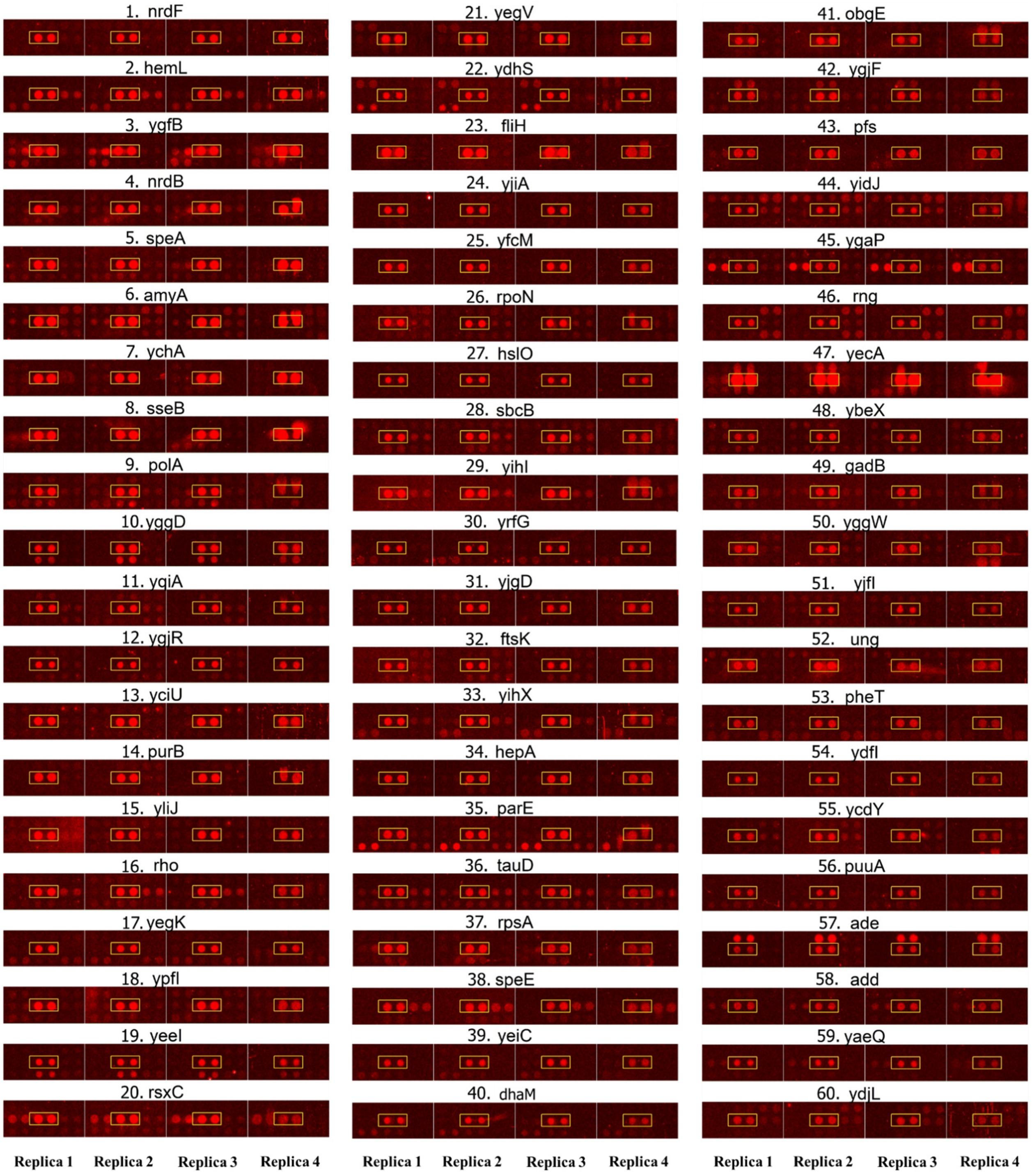

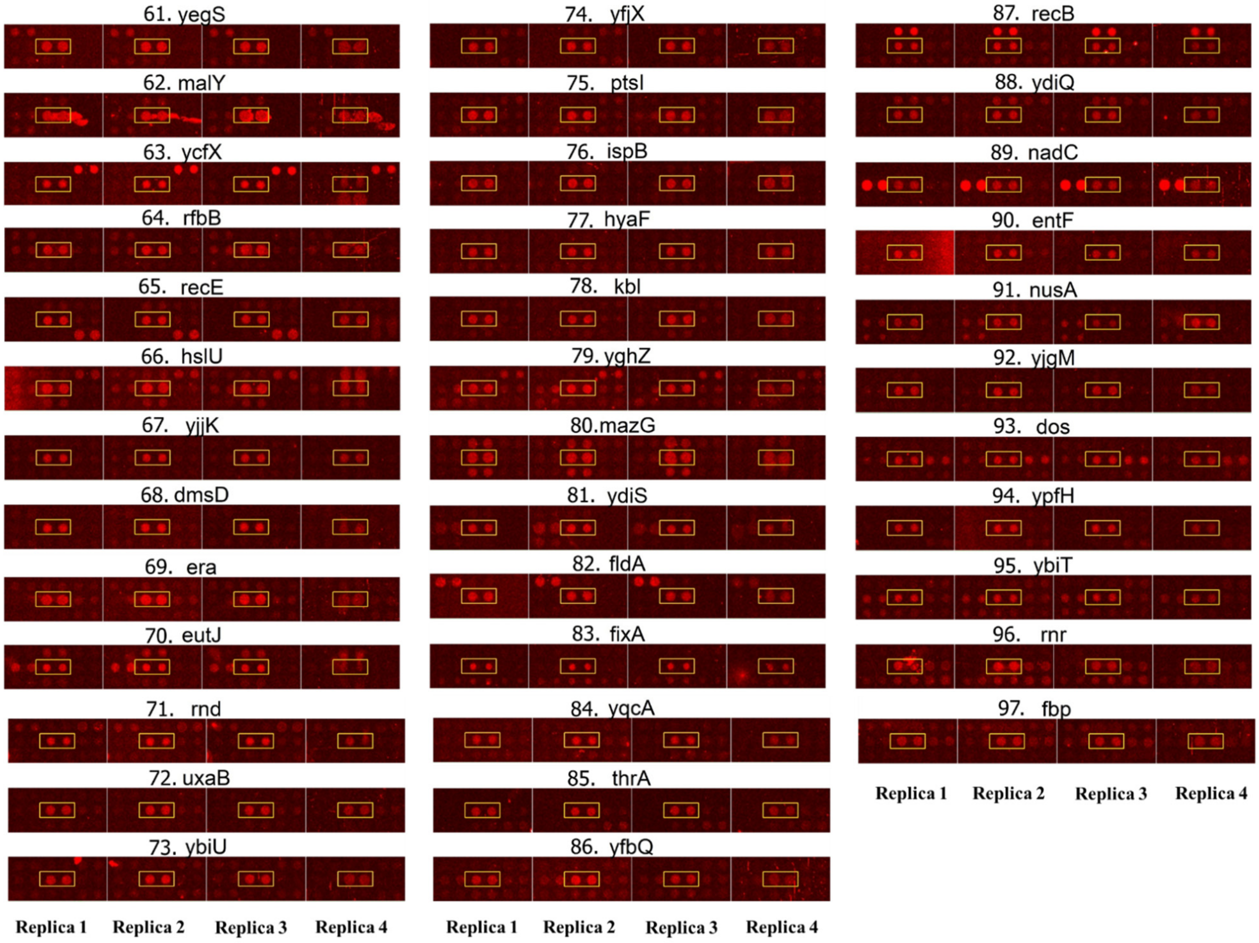

Supplementary Figure S1. Enlarger images of entire protein targets of Polyphemusin-I identified from quadruplicate *Escherichia coli* proteome microarrays. Enlarge image of the identified 97 protein targets of Polyphemusin-I on quadruplicate *Escherichia coli* proteome microarrays (right). Each red spot (in duplicate) inside square box represent the individual protein target of Polyphemusin-I identified individually in *Escherichia coli* proteome microarrays, in total of four replicate. (Four replicated assays of *Escherichia coli* proteome microarrays with Polyphemusin-I are represented by replica 1, 2, 3 and 4, respectively).
